# Milli-electronvolt monochromatization of hard X-rays with a sapphire backscattering monochromator

**DOI:** 10.1107/S090904951102485X

**Published:** 2011-07-20

**Authors:** I. Sergueev, H.-C. Wille, R. P. Hermann, D. Bessas, Yu. V. Shvyd’ko, M. Zając, R. Rüffer

**Affiliations:** a European Synchrotron Radiation Facility, F-38043 Grenoble, France; b Deutsches Elektronen-Synchrotron, D-22607 Hamburg, Germany; c Jülich Center for Neutron Science JCNS and Peter Grünberg Institut PGI, JARA-FIT, Forschungszentrum Jülich GmbH, D-52425 Jülich, Germany; d Faculté des Sciences, Université de Liège, B-4000 Liège, Belgium; e Advanced Photon Source, Argonne National Laboratory, Argonne, IL 60439, USA; f Faculty of Physics and Applied Computer Science, AGH University of Science and Technology, 30-059 Kraków, Poland

**Keywords:** X-ray optics, monochromator, energy resolution, sapphire, backscattering, inelastic scattering

## Abstract

A sapphire backscattering monochromator with 1.1 (1) meV bandwidth for hard X-rays (20–40 keV) is reported. The optical quality of several sapphire crystals has been studied and the best crystal was chosen to work as the monochromator. The small energy bandwidth has been obtained by decreasing the crystal volume impinged upon by the beam and by choosing the crystal part with the best quality. The monochromator was tested at the energies of the nuclear resonances of ^121^Sb at 37.13 keV, ^125^Te at 35.49 keV, ^119^Sn at 23.88 keV, ^149^Sm at 22.50 keV and ^151^Eu at 21.54 keV. For each energy, specific reflections with sapphire temperatures in the 150–300 K region were chosen. Applications to nuclear inelastic scattering with these isotopes are demonstrated.

## Abstract 2

Monochromatization of hard X-rays in the 20–40 keV energy range to ∼1 meV bandwidth using a sapphire backscattering monochromator is demonstrated.

## 1. Introduction

Several methods used for studying structure and dynamics in condensed matter require high-energy-resolution monochromatization of the X-rays. In particular, inelastic X-ray scattering (Burkel, 2000 ▶), nuclear resonant scattering (Gerdau & de Waard, 1999/2000 ▶) and precise measurements of the crystalline lattice parameters (Shvyd’ko *et al.*, 2000 ▶) demand monochromators with a relative energy resolution (Δ*E*/*E*) of 10^−7^–10^−8^ and with maximum throughput.

Most high-energy-resolution measurements are nowadays carried out using monochromatization of synchrotron radiation based on diffraction by silicon crystals for which large ingots of extremely good quality are available. Depending on the application, two different optical schemes are employed.

Experiments where the energy of the X-rays is not determined by the application are normally carried out using a single reflection in backscattering geometry, *i.e.* with a Bragg angle around π/2 (Graeff & Materlik, 1982 ▶; Dorner *et al.*, 1986 ▶). The energy of the reflected X-rays is defined by the interplanar distance of the chosen reflection and can be tuned by variation of the crystal temperature. The angular acceptance of backscattering reflections is larger than the divergence of the incident synchrotron radiation that provides a high throughput of the monochromator. However, in silicon the number of back-reflections with different interplanar distances is small and only a discrete set of X-ray energies can be explored by this type of backscattering monochromator.

Another approach is used for applications where a specific photon energy is required, particularly for nuclear resonance scattering experiments, where the energy is defined by the nuclear transition of a particular isotope. Here, monochromatization is achieved in two steps (for reviews see, for example, Toellner, 2000 ▶; Shvyd’ko, 2004 ▶; Ishikawa *et al.*, 2005*b* ▶). First, the incoming X-ray beam is collimated by one or more asymmetric low-order reflections in order to match the angular acceptance of the high-order reflection, which, in a second step, reflects X-rays in a narrow energy range. Such monochromators allow tunability of the reflected energy in a wide range by variation of the incident angle of reflection and provide monochromatization around or below 1 meV in the 20–30 keV range. However, the throughput of these multiple-crystal Si high-resolution monochromators (HRMs) is limited by the number of reflections with a typical spectral efficiency around 10% (Baron *et al.*, 2001 ▶; Toellner *et al.*, 2006*b* ▶). The throughput can be improved by lowering the temperature of the crystals which increases the Debye–Waller factor and, consequently, increases the reflectivity and the angular acceptance of the high-order reflections as shown by Toellner *et al.* (2006*a* ▶), where a cryogenically cooled Si HRM for 23.88 keV with a bandwidth of 1.3 meV and a spectral efficiency of 37% was presented.

However, for X-ray energies above 30 keV, cooling of the Si crystal down to nitrogen temperature leads to an angular acceptance of the high-order reflections of about 0.1–0.2 µrad, which is two orders of magnitude smaller than the typical divergence of synchrotron radiation at third-generation sources. Such a large mismatch between the divergence of the X-ray beam and the acceptance of the high-order reflections is difficult to overcome without significant diminution of the spectral efficiency. A monochromator for 37.1 keV with a bandwidth of 1.7 meV has been presented (Tsutsui *et al.*, 2007 ▶), for which the Si(4 4 32) reflection at ∼66 K was used. The spectral efficiency is not reported in this work; assuming, however, that no collimating optics was used, the efficiency can be roughly estimated as the ratio of the angular acceptance of the reflection (∼0.2 µrad) and the divergence of the synchrotron beam (∼10 µrad), *i.e.* 2%.

Monochromatization, which combines both high efficiency using backscattering by a single-crystal and free choice of the X-ray energy, can be achieved by backscattering from a sapphire crystal (Shvyd’ko & Gerdau, 1999 ▶; Shvyd’ko, 2004 ▶). The lower symmetry of the sapphire compared with silicon leads to a larger number of different interplanar spacings. As a consequence, several back-reflections with high efficiency and small bandwidth can be found for specific energies above 20 keV by adjusting the temperature of the crystal between 100 and 400 K. A sapphire backscattering monochromator (BSM) has already been applied to observe nuclear resonances of ^161^Dy at 25.61 keV (Shvyd’ko *et al.*, 2001 ▶), ^119^Sn at 23.88 keV, ^151^Eu at 21.54 keV (Shvyd’ko *et al.*, 2002 ▶), ^121^Sb at 37.13 keV (Wille *et al.*, 2006 ▶) and ^125^Te at 35.49 keV (Imai *et al.*, 2007 ▶; Wille *et al.*, 2010 ▶) with bandwidths of more than 4 meV compared with the 1 meV or sub-meV bandwidths expected in theory. Unfortunately, insufficient quality of available sapphire crystals restricts the application of this method for lattice dynamics studies, where an energy resolution of 1 meV or less is highly demanded.

Here we present an approach which allows us to cope with the insufficient quality of available sapphire crystals and to obtain a monochromator for the 20–40 keV energy range with a bandwidth of about 1 meV and reasonably high throughput. Our approach is to decrease the crystal volume impinged upon by the X-ray beam down to the characteristic size of an ideal sub-grain of the sapphire crystal. This was done by using a thin crystal and by focusing and/or limiting the transverse beam size. Ideal spots for monochromatization were found by scanning over the crystal.

The efficiency of the monochromator has been tested using the nuclear resonances of ^121^Sb, ^125^Te, ^119^Sn, ^149^Sm and ^151^Eu with nuclear transition energies between 20 and 40 keV. The experimental resolution for all energies was 1–1.2 meV, with spectral efficiencies between 10 and 65%. The monochromator was used for nuclear inelastic scattering by elemental Sb, Te and β-Sn. The measurements show an efficiency of the monochromator to study the lattice dynamics in compounds containing those elements. For the 20–30 keV energy range the spectral bandwidth of the sapphire BSM is similar to that of the conventional (room-temperature) multiple-crystal Si HRMs; however, the spectral efficiency is higher. For all measurements at different energies the same monochromator and the same crystal has been used. A particular back-reflection for the desired X-ray energy was reached by rotation of the monochromator housing and by temperature variation.

## 2. Choice of the proper sapphire crystal

The X-ray topography characterization (Chen *et al.*, 2001 ▶) of the sapphire crystals grown by different methods shows that dislocations, which spoil the reflectivity, exist in most of the crystals. The orientation of the dislocations and their density depends on the growth method. On the other hand, even within one crystal the density of the dislocations varies significantly, so that regions without dislocations can sometimes be found. As a result, a small defect-free volume can have a backscattering performance beyond the average of the crystal.

Limiting the crystal volume impinged upon by the X-ray beam can be easily performed in the transverse plane by limiting the beam size by slits or by focusing. At the same time the longitudinal size of the crystal part which is involved in the scattering is limited to a few extinction lengths. For the 20–40 keV energy range this size can vary between 1 and 10 mm, which is larger than the defect-free parts of the available crystals. Therefore, thin crystals have to be chosen to restrict the longitudinal component of the scattering volume, so as to reduce mosaicity broadening. However, the reduction of the crystal thickness below a few extinction lengths results in a broadening of the reflection according to the dynamical theory of X-ray scattering. For non-perfect crystals this means a trade-off in the crystal thickness to achieve the best energy resolution, as the presence of defects has been shown to significantly increase the energy width of the reflection (Wille *et al.*, 2006 ▶, 2010 ▶; Imai *et al.*, 2007 ▶).

**Figure 1:**
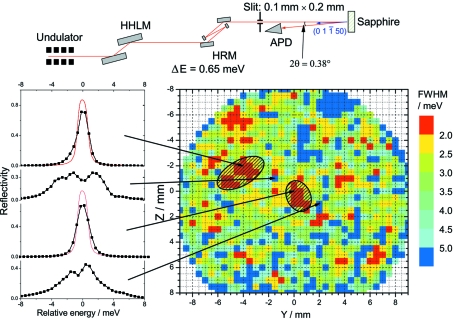
Experimental set-up and results of the sapphire crystal quality characterization. Top: experimental set-up at ID18 with the high-heat-load monochromator (HHLM), high-resolution monochromator (HRM) and Si avalanche photodiode (APD). Bottom right: map of the energy width for the reflection (0 1 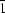 50) at different points on the sapphire crystal. The dashed ellipses denote the parts of the crystal which were used for monochromatization. Bottom left: energy dependence of the reflectivity for the spots on the crystal indicated by the arrows; the red line is the theoretical reflectivity calculated for an ideal HRM and sapphire crystal.

In the first step of this work we have characterized the quality of several sapphire crystals by measuring the energy dependence of the Bragg reflectivity. The experiment was performed at the nuclear resonance beamline ID18 of the European Synchrotron Radiation Facility (Rüffer & Chumakov, 1996 ▶). The experimental set-up is shown in Fig. 1 (top) ▶. The reflection (0 1 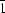 50) (in the hexagonal notation) has been used at an energy of 23.906 keV in almost exact backscattering geometry, with a Bragg angle of 89.81°. The extinction length of this reflection is 145 µm. The energy of the incident radiation was monochromated to 0.65 meV bandwidth and varied in a ±20 meV range around the reflection using a multiple-crystal Si HRM (Chumakov *et al.*, 1998 ▶ 1). The reflected intensity was measured by an avalanche photodiode (Baron *et al.*, 2006 ▶). The incident beam size was reduced to 0.1 mm × 0.2 mm (vertical × horizontal) by slits inserted after the Si HRM. The sapphire crystals were installed onto translation stages and the energy dependence of the reflectivity was studied over the entire crystal with 0.5 mm increments in the horizontal and vertical directions.

Several sapphire crystals produced by different methods and with different thicknesses have been investigated. Reflectivity curves with almost theoretical widths of 1.5 meV were observed for a crystal of thickness 1 mm cut parallel to the (0 0 0 1) plane grown (at the Institute for Single Crystals, Kharkov, Ukraine) by the heat-exchange method (Schmid *et al.*, 1994 ▶). The same crystal also shows the smallest dislocation density in the topography measurements (Shvyd’ko, 2004 ▶). A map of the energy width of the crystal reflectivity taken as the full width at half-maximum (FWHM) is shown in Fig. 1 ▶ together with typical reflectivity curves. Note that the reflectivity curves with large FWHM consist of several peaks. The width of each peak is comparable with the theoretical width and the separation between them is of the order of 1–4 meV. This indicates that the crystal consists of a mosaic of separate sub-grains of almost perfect quality and rather macroscopic size (in the sense that only a few grains are seen in the scattering volume of 0.1 × 0.2 × 1 mm). At the same time the tails of the reflectivity are enhanced compared with theory, probably owing to micro defects which lead to diffuse scattering. This crystal was chosen to be used for the sapphire BSM. In order to restrict the crystal thickness along the beam, reflections which are close to the (0 0 0 1) direction were chosen for each energy.

## 3. Experimental set-up for nuclear inelastic scattering measurements

**Figure 2:**
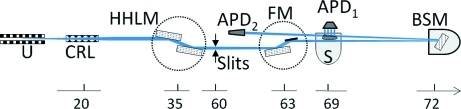
Experimental set-up for nuclear inelastic scattering measurements at ID22N with undulator (U), compound refractive lenses (CRL), high-heat-load monochromator (HHLM), focusing monochromator (FM), sample in the cryostat (S), sapphire crystal in the nitrogen gas cryostat (BSM), avalanche photodiodes detectors in the forward direction (APD_2_) and close to the sample (APD_1_). The bottom scale shows the distances in meters from the source to the optical elements.

The experimental set-up of the monochromator and its application to observe nuclear inelastic scattering was carried out at the nuclear resonance station ID22N of the European Synchrotron Radiation Facility in the 16-bunch timing mode. The experimental set-up is shown in Fig. 2 ▶. The undulator beam was vertically focused using Be compound refractive lenses (Snigirev *et al.*, 1996 ▶) to 7 ± 3 µrad angular spread and monochromated using a Si(1 1 1) high-heat-load monochromator (HHLM) to a bandwidth of 3–7 eV, depending on the incident energy. (i) For energies below 25 keV, a sagittally focusing monochromator consisting of a pair of flat and bent Si(1 1 1) crystals is installed downstream of the HHLM (Freund *et al.*, 1998 ▶). The throughput was 40%, *i.e.* two times smaller than theoretically calculated, owing to non-optimal mounting of the crystals. The beam size at the sapphire crystal position was ∼0.2 mm × 0.2 mm. (ii) At energies above 30 keV the horizontal beam size was defined by slits and the beam size at the sapphire crystal was ∼0.4 mm × 1 mm (vertical × horizontal). Consequently, the flux is reduced to ∼65% of the original beam with 1.5 mm horizontal size (FWHM).

The sapphire crystal, which acts as a BSM, scattered the beam up in the vertical plane with an angular offset of 0.10° between the incident and reflected beams. The crystal was located in a nitrogen gas flow cryostat (van der Linden *et al.*, 2007 ▶) with a typical flow rate of 0.2–0.6 l min^−1^. The temperature of the gas was controlled by a heater with 1 mK precision. The temperature of the sapphire crystal was measured by a calibrated PT100 temperature sensor installed in the gas close to the crystal (at 1 mm distance). The nuclear inelastic scattering experiments were carried out by scanning the temperature of the sapphire crystal ∼1 K around the temperature corresponding to the nuclear resonance energy, called here the resonance temperature, with a typical rate of 1 K h^−1^. The cryostat was installed on a two-circle diffractometer and translation stages in order to adjust particular reflections and to optimize the position of the beam on the crystal. The photon flux was measured by three ionization chambers installed after the high-heat-load, focusing and backscattering monochromators, respectively.

The samples were installed in a close-cycle cryostat around 3 m from the sapphire crystal, resulting in about the same beam size on the sample and on the crystal. Avalanche photodiode detectors (Baron *et al.*, 2006 ▶) allowed the delayed nuclear resonant scattered signal to be separated from the electronic scattering. A detector close to the sample measured the products of nuclear inelastic absorption. A detector installed in the forward direction measures the nuclear forward scattering (NFS). Owing to the elastic nature of NFS and the small energy width of the nuclear excited states (≤1 µeV), the detection of the NFS signal enables a direct measurement of the sapphire BSM instrumental function.

## 4. Nuclear inelastic scattering with different isotopes

The performance of the sapphire BSM was tested by measuring nuclear resonance scattering on several Mössbauer isotopes with resonance energies between 20 and 40 keV. The absolute energy of the nuclear resonance is determined *via* the sapphire lattice parameters at the resonance temperature. The energy scale of the nuclear inelastic scattering is proportional to the temperature variation around the resonance temperature with the lattice thermal expansion coefficient as proportionality coefficient. The sapphire thermal expansion coefficients have been measured by different methods (White & Roberts, 1983 ▶; Lucht *et al.*, 2003 ▶) and deviate by up to 5%. In this work, for the energy calibration, we have used the dilatometry data on the thermal expansivity obtained by White & Roberts (1983 ▶).

The absolute energy of the nuclear resonances was calculated from resonance temperature using the absolute value of the sapphire lattice parameters at room temperature (Shvyd’ko *et al.*, 2002 ▶) and the variation of the lattice with temperature (White & Roberts, 1983 ▶; Burghartz & Schulz, 1994 ▶; Lucht *et al.*, 2003 ▶). From a comparison of these results the absolute error on the energy is estimated as 0.5 eV.

### 4.1. Nuclear resonance with ^121^Sb at 37.13 keV

Backscattering reflections in sapphire crystal with a spectral efficiency of more than 50% which match the 37.13 keV energy of the nuclear transition in ^121^Sb for a sapphire temperature between 150 and 300 K are presented in Table 1 ▶. We have chosen the (8 16 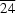 40) reflection which is inclined by 59.1° to the (0 0 0 1) direction leading to ∼2 mm crystal thickness along the beam. For this crystal thickness the expected theoretical resolution is 0.4 meV and the spectral efficiency of the reflection is about 60%, as shown in Table 2 ▶. In principle, the scattering vector of the (8 13 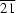 52) reflection is even closer to (0 0 0 1); however, the larger extinction length leads to an efficiency three times smaller.

**Figure 3:**
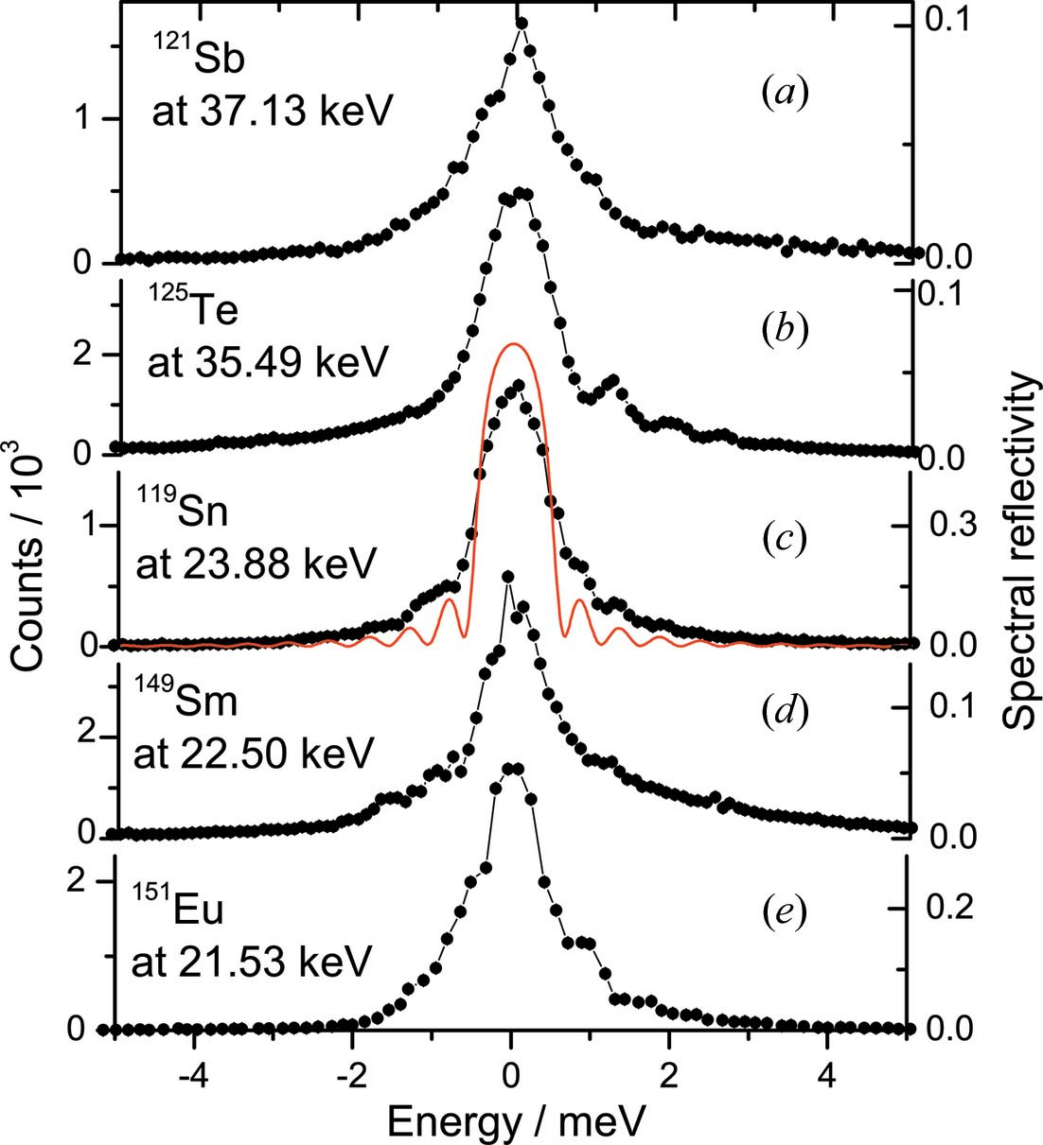
Instrumental functions of the sapphire backscattering monochromator measured with NFS of several Mössbauer isotopes at the indicated energies. The corresponding reflections are presented in Table 2 ▶. The thin red line in (*c*) shows the theoretical simulation.

The nuclear resonance of ^121^Sb was found at the sapphire temperature of 236.8 (1) K which corresponds to an energy of 37.1292 (5) keV. This value is consistent with the previously reported energy of 37.1298 (2) keV (Wille *et al.*, 2006 ▶). Fig. 3(*a*) ▶ shows the instrumental function measured by the elemental Sb NFS upon temperature variation of the sapphire crystal. The instrumental function has an energy bandwidth (FWHM) of 1.2 meV, a maximum spectral efficiency of about 10% and a total flux after the BSM of about 4.5 × 10^7^ photons s^−1^. The spectral efficiency is derived as the ratio of the flux per meV before and after the BSM. The incoming flux per meV is calculated as the ratio of the total flux to the energy bandwidth of the HHLM. The flux per meV after the BSM is calculated from the total flux and the exact shape of the instrumental function. The measured energy bandwidth is three times larger and the spectral efficiency is six times smaller than the theoretically expected values. This is probably related to the large scattering volume of the crystal. Although the energy bandwidth is three times larger than expected, it is smaller than the previously reported values of 4.5 meV (Wille *et al.*, 2007 ▶) and 1.7 meV (Tsutsui *et al.*, 2007 ▶).

**Figure 4:**
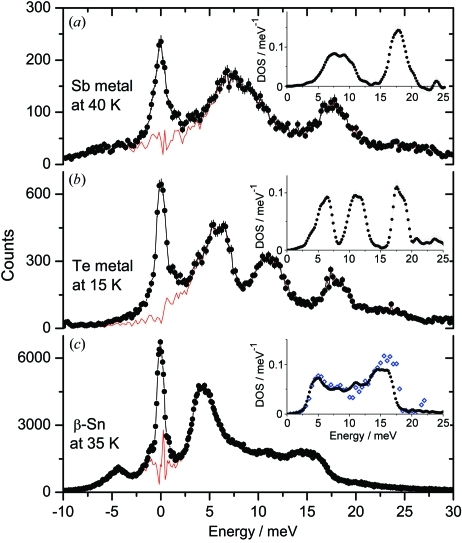
Nuclear inelastic scattering measured with the ^121^Sb, ^125^Te and ^119^Sn nuclear resonances. The red lines show the inelastic spectra with subtracted elastic lines. Insets show the phonon densities of states derived from these data. The blue open diamonds in the inset of (*c*) denote the phonon density of states of β-Sn obtained by Si HRM at 100 K (Barla *et al.*, 2000 ▶).

The nuclear inelastic scattering spectrum for elemental Sb with natural isotopic abundance is shown in Fig. 4(*a*) ▶. The measurements were performed by repetitively scanning up and down the sapphire temperature for about 2 h. The phonon density of states derived from this spectrum is shown in Fig. 4(*a*) ▶. It is consistent with the results obtained by inelastic neutron scattering (Salgado, 1974 ▶; see also Schober & Dederichs, 1981 ▶).

### 4.2. Nuclear resonance with ^125^Te at 35.49 keV

Several Bragg reflections that match the ^125^Te resonance energy (*E* = 35.49 keV) are presented in Table 1 ▶. Among them we have chosen the (9 1 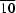 68) reflection which has the smallest angle relative to the (0 0 0 1) direction and the smallest (∼1 mm) crystal thickness along the beam. The nuclear resonance signal of ^125^Te has been observed at 219.5 (1) K, which corresponds to an energy of 35.4920 (5) keV. Previous measurements with the same reflection report a resonance temperature of 218 K (Imai *et al.*, 2007 ▶) which is 1.5 K below our result. This discrepancy could be due to the heat load effect, which will be discussed later.

The measured instrumental function shown in Fig. 3(*b*) ▶ has an energy bandwidth (FWHM) of 1.1 meV and a spectral efficiency of 16%. The total flux after the BSM was about 7.5 × 10^7^ photons s^−1^. This energy resolution is still twice as worse as the theoretical one, but it is the best experimentally observed resolution in the 35–40 keV energy range. We have measured the nuclear inelastic scattering on elemental Te with 93% ^125^Te enrichment. The spectrum was obtained within 2 h and the derived phonon density of states are shown in Fig. 3(*b*) ▶.

### 4.3. Nuclear resonances at 21–24 keV

The efficiency of our HRM has also been tested with nuclear resonances located in the 21–24 keV energy range: ^119^Sn at 23.88 keV, ^149^Sm at 22.50 keV and ^151^Eu at 21.54 keV. The reflections for each energy (see Table 2 ▶) have been chosen to have the smallest thickness of the crystal along the beam. The temperatures (energies) where resonance signals have been observed agree well with the values previously reported (Kikuta, 1994 ▶; Koyama *et al.*, 1996 ▶; Leupold *et al.*, 1996 ▶; Shvyd’ko *et al.*, 2002 ▶).

For the ^149^Sm nuclear resonance we obtained an energy of 22.5015 (5) keV. This energy is close to the statistical average of the previously reported values of 22.494 (11) keV (Antman *et al.*, 1970 ▶) and 22.519 (8) keV (Meyer *et al.*, 1982 ▶) but has ten times better accuracy.

The instrumental functions measured by NFS are shown in Figs. 3(*c*), 3(*d*) and 3(*e*) ▶. The measured energy bandwidths (FWHM), spectral efficiencies and total flux are presented in Table 2 ▶. A reflection with close to theoretical properties is observed for ^119^Sn at 23.88 keV with a spectral efficiency of 65%. The obtained energy bandwidth of 1.1 meV is acceptable for carrying out efficient measurements of the phonon dynamics as shown in Fig. 4(*c*) ▶ where the nuclear inelastic scattering spectrum of β-Sn and the derived phonon density of states are shown together with the phonon density of states obtained using multiple-crystal Si HRM (Barla *et al.*, 2000 ▶).

On the other hand the spectral efficiency of 20% and the energy bandwidth of 1.0 meV of the back-reflection (5 10 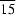 22) used for ^149^Sm at 22.50 keV is two to three times worse than the theoretical predictions, as seen in Table 2 ▶. This discrepancy, as well as the good agreement of the experimental and theoretical characteristics of the (4 4 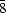 45) reflection used for ^119^Sn, correlates with the difference in the crystal thicknesses along the beam (2.1 and 1.1 mm) and with the difference in the extinction length (0.46 and 0.28 mm).

## 5. Discussion

Our study reveals that a high-resolution monochromator based on backscattering geometry with a sapphire crystal can provide an energy bandwidth of 1–1.2 meV (FWHM) and a spectral efficiency higher than 10% for all chosen energies (reflections) between 20 and 40 keV, even with a non-perfect crystal. However, whereas characteristics of the reflection (4 4 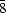 45) at 23.88 keV are in good agreement with theory, the reflection (8 16 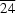 40) at 37.13 keV provides an energy resolution three times broader and a spectral efficiency six times smaller than theoretically expected. All other reflections show results in between these extreme cases. The obvious explanation of this effect comes from the correlation of the crystal volume impinged upon by the beam and the quality of the reflection. The measurements at 23.88 keV have been performed with a beam size on the sapphire crystal of 0.2 mm × 0.2 mm and with a crystal thickness of ∼1 mm. On the other hand the measurements at 37.13 keV have been performed with a beam size of 0.4 mm × 1 mm and with a crystal thickness of ∼2 mm. We believe the factor of 20 in the scattering volume, combined with the difference in the extinction length, explain the difference in the reflection quality.

**Figure 5:**
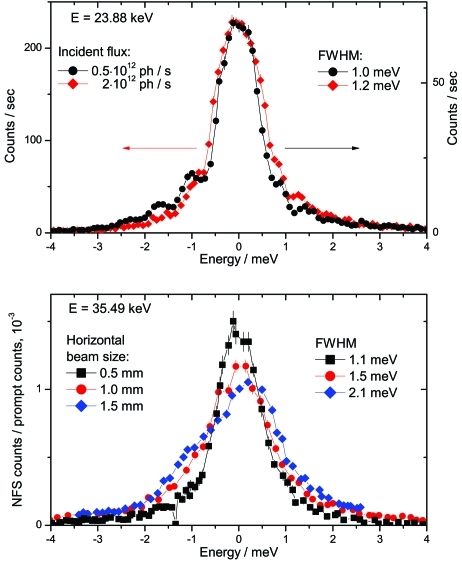
Dependence of the instrumental function shape on the heat load (top) and the horizontal beam size (bottom) measured by NFS with ^119^Sn at 23.88 keV (top) and ^125^Te at 35.49 keV (bottom). In the bottom panel the NFS count rate is normalized to the electronic (prompt) scattering in the inelastic detector.

Another important factor which influences the quality of the reflection is the heat load which produces a temperature gradient in the sapphire crystal. The influence of the heat load on the monochromator performance was studied at 23.88 keV with the ^119^Sn nuclear resonance. The effect of the heat load is expected to be highest at this energy because of the high incident photon flux and the smallest beam size on the sapphire crystal. The instrumental functions measured with and without an absorber, which attenuates the photon flux by a factor of four, are presented in Fig. 5 ▶. For an incident flux of 5 × 10^11^ photons s^−1^, corresponding to 1 mW absorbed power in the sapphire crystal, the reflectivity curve is essentially similar to the theoretical curve and the heat load effects are negligible. Increasing the flux to 2 × 10^12^ photons s^−1^ (4 mW absorbed power) results in a broadening of the reflectivity curve by 0.2 meV and in a smoothing of the oscillations on the tails. Thus, the heat load has some influence on the energy resolution which is not critical for the current set-up. In general, this problem can be solved by introducing a post-monochromator with an intermediate resolution between the HHLM and the sapphire BSM, similar to what is done for the backscattering with Si crystals. In our case the focusing monochromator can be used as the post-monochromator using, for example, the Si (3 1 1) reflections.

The effect of the size of the beam footprint on the crystal has been studied with 35.49 keV photons at the ^125^Te nuclear resonance. The instrumental functions shown in Fig. 5 (bottom) ▶ are measured with a vertical beam size of 0.4 mm and horizontal beam sizes of 0.5, 1 and 1.5 mm. A pronounced degradation of the energy resolution from 1.1 meV to 2.1 meV is observed. The heat load cannot explain such degradation since the effect is much more pronounced than in the previous case while the absorbed power (0.6–2 mW) is smaller at this energy. In addition, the instrumental function becomes asymmetric, probably due to illumination of the crystal parts with slightly mismatched lattice constants. Thus, the increase of the beam spot size significantly influences the performance of the monochromator with non-ideal sapphire crystals.

For nuclear inelastic scattering experiments with the sapphire BSM the measurement of the sapphire temperature with mK precision is a crucial point. In the current set-up the temperature sensor was installed in the nitrogen gas measuring the temperature of the sapphire *via* convection heat exchange between the gas and the crystal. Scanning the temperature up and down with a typical rate of 1 K h^−1^, we did not find any difference in the shape of the nuclear inelastic spectra and the resolution functions, which shows that such a rate is slow enough to achieve thermal stability in the cryostat. On the other hand we found that the temperature where the elastic nuclear resonance scattering has been observed depends linearly on the incoming flux. The proportionality coefficient was obtained for each reflection and was taken into account in the energy calibration. The temperature difference between the sensor and the scattering part of the sapphire crystal was about 50 mK per 1 mW of absorbed power for all energies.

The measured nuclear inelastic spectra from the samples with isotopes of ^119^Sn, ^149^Sm and ^151^Eu were compared with the spectra measured using multiple-crystal Si HRMs (Chumakov *et al.*, 1998 ▶; Barla *et al.*, 2004 ▶) and showed coincidences in the limit of 5% in the energy calibration. The discrepancy is probably related to uncertainty in the thermal expansion coefficients.

**Figure 6:**
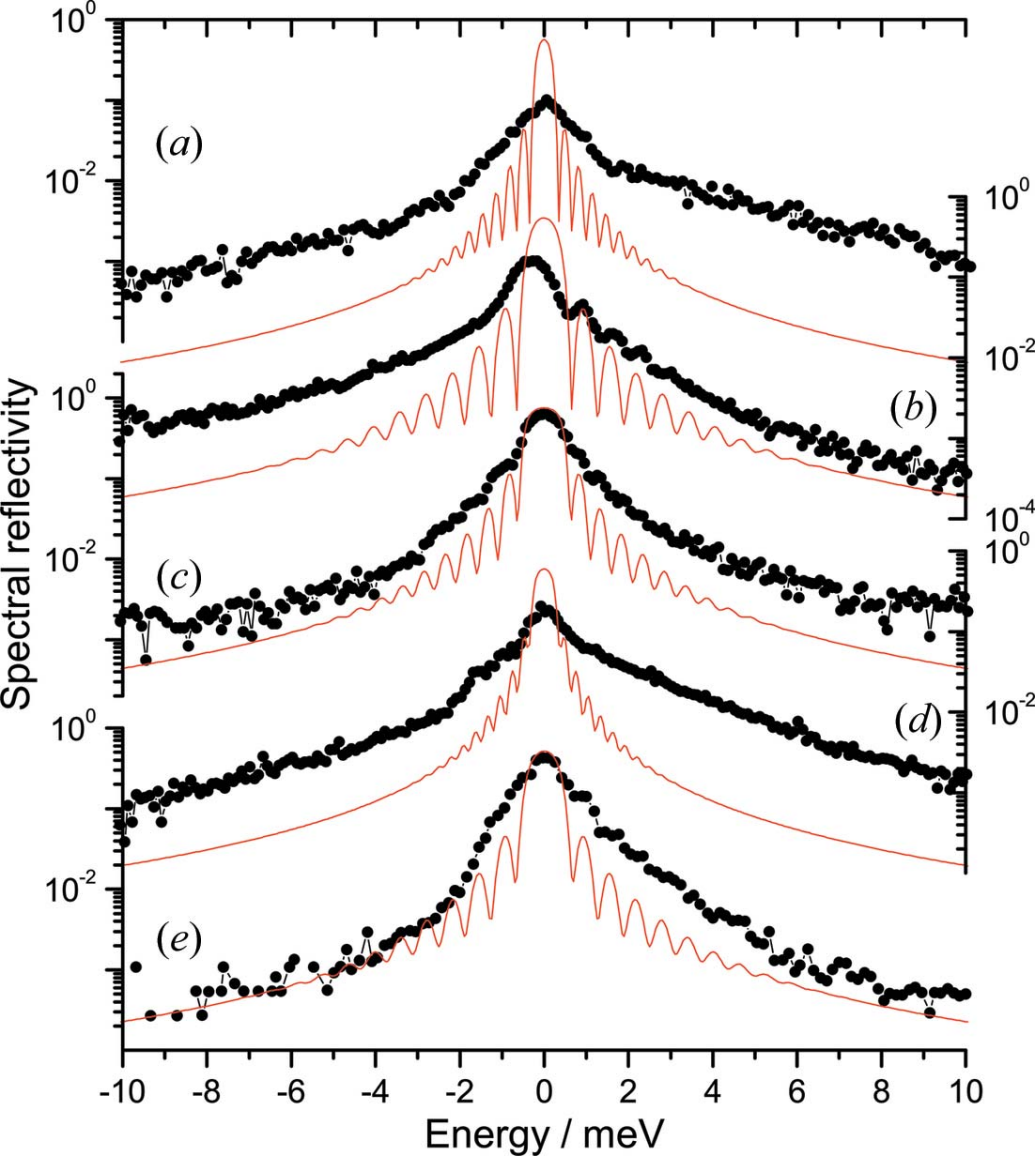
Instrumental functions of the sapphire backscattering monochromator measured with NFS presented on a logarithmic scale. The data are scaled to have maximum spectral efficiency according to Table 2 ▶. (*a*)–(*e*) denote the same isotopes as in Fig. 3 ▶. The thin grey lines (red online) show simulation according to theory with the crystal thicknesses indicated in Table 2 ▶.

Besides the energy bandwidth (FWHM) of the resolution function, the tails are important for the practical use of the monochromator. Fig. 6 ▶ shows the instrumental functions measured by NFS (on a logarithmic scale) for the tested nuclear resonances as compared with the theoretical simulation of the reflectivity. The tails follow the *E* ^−2^ energy dependence similar to the theoretical curves. However, they are enhanced in magnitude, probably because of diffuse scattering on defects in the sapphire. The main factor affecting the magnitude of this enhancement in the tails is the crystal thickness along the beam for the chosen reflections. A clear difference is seen for the tail enhancement for ∼1 mm and ∼2 mm crystal thicknesses in Fig. 6 ▶. This observation emphasizes the importance of limiting the thickness of the sapphire crystal used as monochromator.

The enhanced tails of the instrumental function may complicate subtraction of the elastic peak from the inelastic spectrum and, as a consequence, the correct information about the low-energy region of the phonon density of states. However, this problem is also related to the relative strength of the scattering in the elastic and inelastic channels of the nuclear resonant scattering which depends on the properties (internal conversion coefficient, energy of emitted photons and characteristic Lamb–Mössbauer factor) of the specific isotope. In particular, for ^119^Sn, ^125^Te and ^121^Sb the signal in the elastic channel is rather weak. The influence of the elastic peak on the inelastic spectrum becomes negligible above 2–3 meV and the phonon density of states is reliable starting from this energy, as seen from Fig. 4 ▶. On the other hand, for ^149^Sm the signal in the elastic channel is typically much higher than in the inelastic one and the reliable region of the phonon density of states starts above 5 meV.

As compared with the multiple-crystal Si HRM, the tails of the sapphire BSM are enhanced even in the ideal theoretical case, which is a consequence of the single *versus* multiple Bragg reflections. As a result the features in the phonon DOS are typically broadened. This problem can be solved by the partial deconvolution of the phonon DOS with the instrumental function (Kohn & Chumakov, 2000 ▶).

## 6. Conclusion

In summary, we have demonstrated a versatile high-resolution monochromator based on backscattering from sapphire crystals working in the energy range 20–40 keV with an energy bandwidth of ∼1 meV and with high throughput. The concept of such a monochromator has been suggested by Shvyd’ko & Gerdau (1999 ▶) but the implementation suffered from the quality of available sapphire crystals (Shvyd’ko *et al.*, 2001 ▶; Wille *et al.*, 2006 ▶, 2010 ▶; Imai *et al.*, 2007 ▶). Improvement in the monochromator performance was obtained by decreasing the crystal volume impinged upon by the beam to a size comparable with a characteristic concentration of defects in the crystal. This allows one to choose a part of the crystal with a minimal number of defects. The efficiency of the monochromator is not the same for all energies and depends on the chosen reflection. The feasibility of nuclear inelastic scattering measurements with ^121^Sb, ^125^Te, ^119^Sn, ^149^Sm and ^151^Eu was demonstrated. The partial phonon density of states of these elements can thus be measured for compounds of current scientific interest with high resolution and flux. For ^125^Te at 35.49 keV an energy resolution Δ*E*/*E* = 3 × 10^−8^ was obtained for the first time.

As compared with multiple-crystal Si HRMs the backscattering monochromator has some advantages. The universality of this monochromator allows easy transformation from one energy to another which becomes important with the increase of the number of Mössbauer isotopes studied by nuclear inelastic scattering. Example applications include phase-change materials (Wuttig & Yamada, 2007 ▶) composed of Sb and Te or thermoelectric materials such as filled skutterudites (Snyder & Toberer, 2008 ▶) composed of rare earths and Sb where phonon dynamics of each particular element is of importance. Another advantage of such a monochromator is the spectral efficiency, which is higher than the efficiency of conventional (room-temperature) multiple-crystal Si HRMs. The increased efficiency allows one to measure compounds with a natural isotope abundance which are easily available.

The drawbacks of backscattering monochromators and, in particular, this sapphire monochromator are related to the enhanced tail of the instrumental function which complicates the extraction of the tiny features of the phonon dynamics; in particular, the precise estimation of the Debye energy for some elements may be difficult. Another drawback compared with the inline multiple-crystal Si HRM is the backscattering geometry which puts some restrictions on the sample environment; herein, a distance of 5–7 mm between the direct and backscattered beam was used.

Major improvement of the monochromator efficiency would be achieved by improving the quality of sapphire crystals. This would allow for a significant increase in the efficiency and a decrease of the energy bandwidth. It also would extend the applicability of this monochromator above 40 keV. On the other hand, even with the existing sapphire crystals, improving the monochromator properties can be achieved by improving the efficiency of the transversal focusing and implementing this focusing above 30 keV. Besides the Mössbauer isotopes studied in this work, one can apply the monochromator to carry out nuclear inelastic scattering measurements with ^161^Dy at 25.66 keV (Chumakov *et al.*, 2001 ▶), ^201^Hg at 26.27 keV (Ishikawa *et al.*, 2005*a* ▶), ^189^Os at 36.18 keV and ^129^Xe at 39.58 keV.

## Figures and Tables

**Table 1:** Sapphire backscattering reflections that match the nuclear transition energy in ^121^Sb and ^125^Te All reflections with spectral efficiency above 50% are shown. For each reflection the expected crystal temperature *T*, energy bandwidth Δ*E*, spectral efficiency *R*, extinction length *d*_ext_ and angle ϕ between the diffraction vector of the chosen reflection and (0 0 0 1) are shown. The calculations were performed using the dynamical theory of X-ray diffraction with the sapphire crystal data (Shvyd’ko, 2004 ▶) in the thick crystal approximation.

Reflection (*hkil*)	*T* (K)	Δ*E* (meV)	*R* (%)	*d*_ext_ (mm)	ϕ (°)
^121^Sb, *E* = 37.1292 keV					
(8 13 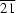 52)	258	0.14	73	1.48	48.1
(7 20 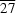 14)	146	0.33	87	0.63	79.6
(15 13 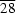 14)	146	0.42	90	0.50	79.6
(21 4 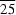 26)	237	0.22	81	0.99	70.5
(8 16 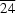 40)	237	0.25	84	0.85	59.1

^125^Te, *E* = 35.4920 keV					
(15 1 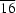 56)	263	0.08	51	2.70	41.1
(20 6 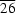 2)	211	0.24	80	0.90	88.5
(5 6 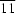 68)	219	0.36	86	0.60	23.9
(9 1 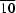 68)	219	0.44	89	0.48	23.9

**Table 2:** Properties of the sapphire backscattering monochromator used in this work to match the nuclear transition energy in ^121^Sb, ^125^Te, ^119^Sn, ^149^Sm and ^151^Eu For each reflection the table provides the measured resonance temperature *T*, the corresponding resonance energy *E*, the extinction length *z*, the variation of the reflection energy with the crystal temperature Δ*E*/Δ*T*, the thickness of the crystal along the beam *d*, the theoretical Δ*E*_th_ and measured Δ*E*_exp_ energy bandwidth (FWHM), the theoretical *R*_th_ and measured *R*_exp_ spectral efficiency, and the measured incident *N*_inc_ and reflected *N*_ref_ flux of the sapphire monochromator at 70 mA storage-ring current.

	Isotope				
	^121^Sb	^125^Te	^119^Sn	^149^Sm	^151^Eu
Reflection	(8 16 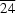 40)	(9 1 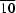 68)	(4 4 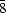 45)	(5 10 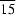 22)	(3 2 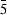 43)
*T* (K)	236.8 (1)	219.5 (1)	192.8 (1)	251.5 (1)	289.1 (1)
*E* (keV)	37.1292 (5)	35.4920 (5)	23.8793 (5)	22.5015 (5)	21.5412 (5)
*z* (mm)	0.85	0.48	0.28	0.46	0.37
Δ*E*/Δ*T* (meV mK^−1^)	−0.156	−0.149	−0.084	−0.100	−0.122
*d* (mm)	1.9 (1)	1.1 (1)	1.1 (1)	2.1 (1)	1.1 (1)
Δ*E*_th_ (meV)	0.39	0.70	0.95	0.52	0.77
Δ*E*_exp_ (meV)	1.2 (1)	1.1 (1)	1.1 (1)	1.0 (1)	1.1 (1)
*R*_th_ (%)	59	60	75	59	55
*R*_exp_ (%)	10	16	65	20	43
*N*_inc_	0.8 × 10^12^	1.5 × 10^12^	2.2 × 10^12^	2.7 × 10^12^	3.4 × 10^12^
*N*_ref_	3.9 × 10^7^	7.7 × 10^7^	7.7 × 10^8^	4.4 × 10^8^	7.4 × 10^8^

## Footnotes

1 An energy resolution of 0.97 meV (FWHM) is reported by Chumakov *et al.* (1998 ▶). Using beam collimation with compound refractive lenses it was possible to obtain a resolution of 0.65 meV, which is close to the theoretical expectation for this multiple-crystal Si HRM.
